# Nanostructured Color Filters: A Review of Recent Developments

**DOI:** 10.3390/nano10081554

**Published:** 2020-08-07

**Authors:** Ayesha Shaukat, Frazer Noble, Khalid Mahmood Arif

**Affiliations:** 1 Department of Mechanical and Electrical Engineering, SF&AT, Massey University, Auckland 0632, New Zealand; a.shaukat@massey.ac.nz (A.S.); f.k.noble@massey.ac.nz (F.N.); 2 Engineering and Management Sciences, Balochistan University of Information Technology, Quetta 87100, Pakistan

**Keywords:** color filters, photonic crystals, nanoholes, mie scattering, liquid crystal display

## Abstract

Color plays an important role in human life: without it life would be dull and monochromatic. Printing color with distinct characteristics, like hue, brightness and saturation, and high resolution, are the main characteristic of image sensing devices. A flexible design of color filter is also desired for angle insensitivity and independence of direction of polarization of incident light. Furthermore, it is important that the designed filter be compatible with the image sensing devices in terms of technology and size. Therefore, color filter requires special care in its design, operation and integration. In this paper, we present a comprehensive review of nanostructured color filter designs described to date and evaluate them in terms of their performance.

## 1. Introduction

The incoming electromagnetic waves of the visible spectrum (400–700 nm) are capable of stimulating the photo-receptor cells of the human retina, which enables color perception. In image sensing devices (cameras, scanners, printers, etc.), which replicate the eye operation, color recognition plays an imperative part to store accurate color information at each pixel of the captured image. The produced color quality via filters is subject to full wave half width maximum value, efficiency and distinct location of transmission peak/reflection dip in the visible spectrum. Hence, all aforementioned characteristics play role in positioning the output color on the CIE Map 1931 [1].

Typically, additive or subtractive color and Bayer filter [2], along with polymer-based filter technologies, have been exploited for image sensing. Over the last three decades, synthetic dyes and pigment-based colors [3] have been extensively used for decorating objects, printing ink, synthetic fibers and identification [4,5,6]. Nevertheless, these filters have many disadvantages such as non-recyclability, non-affordability, and cause environmental damage. Likewise, their performance as image sensors [7,8] also has limitations such as inadequate resolution and fragility. However, recent progress in color filter design, along with the evolution in optical physics [9,10] and fabrication technology [11,12,13], has revolutionized color filtering process and replaced the conventional dry/pigment-based coloring, due to its color tunability, durability, high resolution and environmental friendliness.

In the past Charged Coupled Devices (CCD) were mainly used for different image sensing devices like cameras, microscopes and detectors in the field of life sciences, astronomy and optical microscopy. However, in recent times, Complementary Metal Oxide Semiconductor (CMOS) based sensing devices have out-dated their predecessors in performance by demonstrating low power consumption and higher miniaturization capability.

Today, nano-structural color filters are being designed on a nano-scale and are one of the leading participants in the miniaturization race. The idea of nonstructural color filters is derived from examples in nature, such as butterflies, peacocks, beetles and Pollia fruits [14,15,16]. In structural filters the working principle is based on the light-matter interaction, where light interacts with the structures designed at nanoscale, and allows color filtration via light diffraction, scattering, reflection, refraction and transmission at resonant wavelength.

This review focuses on working principles of nanostructure color filters and absorbers based on Fabry-Ṕerot (FP) cavity thin films, plasmonics, dielectric and liquid crystal technologies. The significant work in these fields is evaluated in terms of color quality, spatial resolution, dependence of output on viewing angle and light polarization, and the required fabrication steps. The details of the dynamic color filters such as mechanically [17], chemically [18] or electrically tuned [19], etc., are beyond the scope of this review. The reader is referred to [20,21,22] for review articles related to these areas.

## 2. Thin Films Color Filters

**Figure 1:**
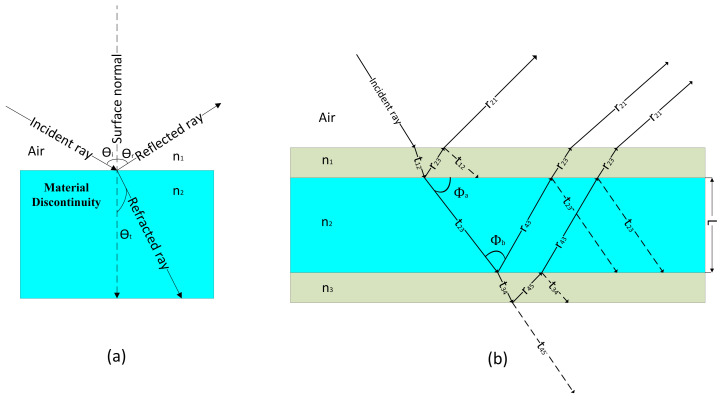
Vector diagram for transmission and reflection in FP-cavity when light comes across (**a**) Single and (**b**) Multiple material discontinuities.

Figure 1a illustrates that a material discontinuity in the propagation path of light from refractive index $n_{1}$ to refractive index $n_{2}$ allows light reflection, refraction and transmission and is dependent on the incident angle $\theta_{i}$.

The Fresnel equations for transmission (*t*) and reflection (*r*) at a material interface for *s* and *p* polarization are given as follows:

(1) $$t_{s}=\frac{2n_{1}cos\theta_{i}}{n_{1}cos\theta_{i}+n_{2}cos\theta_{t}},$$

(2) $$t_{p}=\frac{2n_{1}cos\theta_{i}}{n_{2}cos\theta_{i}+n_{1}cos\theta_{t}},$$

(3) $$r_{s}=\frac{n_{1}cos\theta_{i}+n_{2}cos\theta_{t}}{n_{1}cos\theta_{i}+n_{2}cos\theta_{t}},$$

(4) $$r_{p}=\frac{n_{2}cos\theta_{i}-n_{1}cos\theta_{t}}{n_{2}cos\theta_{i}+n_{1}cos\theta_{t}}.$$

Here, *s* and *p* subscripts indicate reflection *r* and transmission *t* in *s* and *p* polarization, respectively.

In another case, where light strikes a stack of high refractive index layer $n_{2}$ of thickness *L* sandwiched between two layers with refractive indices $n_{1}$ and $n_{2}$ as shown in Figure 1b. This setup is referred as Fabry Ṕerot (FP) interferometer and allows filtration depending on the FP cavity thickness and refractive indices $n_{1}$, $n_{2}$, and $n_{3}$. Here, $r_{ij}$ indicates reflection coefficient at the interface between region 1 and 2.

At FP resonance light gets trapped into the cavity in the form of standing waves and would experience back and forth reflection between two boundaries. On the other hands, the rays propagating in to the regions adjacent to the films will result in total reflection or transmission from the system. Since these rays travel a different distance within the films, they may have destructive or constructive interference. In such case, the intensity of the reflected and refracted waves, along with the refractive index of material, depends on the cavity thickness *L* and results in filtering out the undesired frequencies. Here L=mλ/2n, where *m* is an integer and λ is the wavelength in free space [23,24,25].

FP-based filtration is a multifaceted setup for manipulating the light propagation. It allows certain frequencies to pass through it or can act as an anti reflective (AR) coating to reduce the light reflection in a design, which may cause destructive interference. There have been numerous applications of FP resonators in different science fields such as, clinical diagnosis [26,27], optical communication [28,29] and sensing [30,31,32]. The application of Metal Insulator Metal (MIM)-based FP interferometers, which exhibit color filtration by tuning cavity thickness, sandwiched between two metallic mirror layers is still widely adopted because of its cost-effectiveness, simple design, few fabrication steps [33,34,35,36,37], and easy integration. In this section, the research done to date on FP-based color filters is discussed and reviewed.

A transmission based color filter was designed to filter additive colors (red, green and blue) colors [38]. It comprised of silver (Ag) as metallic layers and silicon dioxide (SiO${}_{2}$) as dielectric. The devices of different dielectric thicknesses *h* were fabricated by using Plasma Enhanced Chemical Vapor Deposition (PECVD) [13] methodology. The device was further evaluated in terms of its polarization sensitivity for oblique incidence, angular dependence, and positional dependence. The minimum reported bandwidth was approximately 100 nm for green color, and the color intensity was 60%. However, for the oblique light incidence the angular dependence of the relative transmission was up to $\approx 12^{\circ }$, and the device was insensitive.

**Figure 2:**
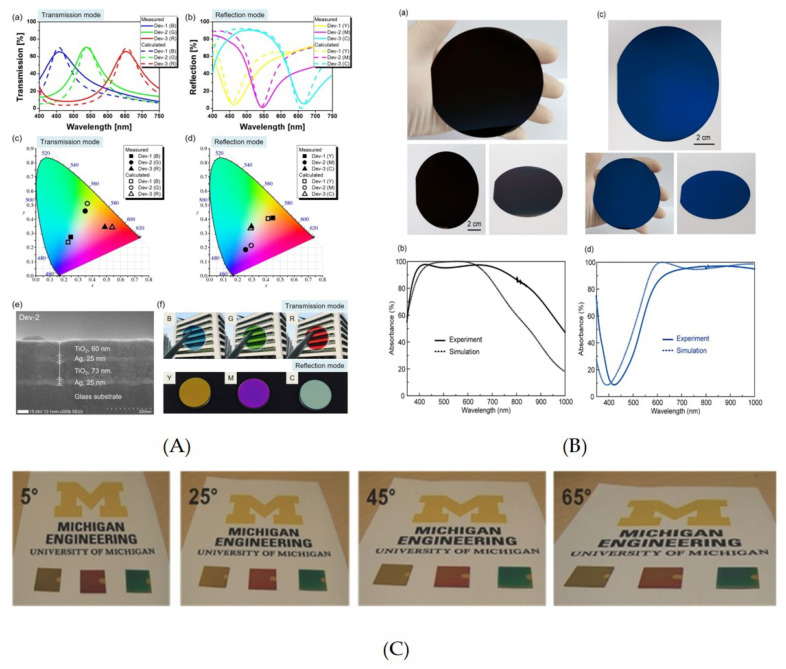
(**A**) Calculated and measured transmission and reflection spectra for the three fabricated devices shows RGB in transmission and CMY in reflection mode. The CIE 1931 further provides the coordinates of the produced results [12]. (**B**) Calculated and measured spectra along with the camera images of broadband visible and near-infrared absorbers implemented with planar nanolayered stacks. Reproduced with permission of [42]. Copyright American Chemical Society, 2020. (**C**) Demonstration of angle invariance of up to 0 to 65${}^{\circ }$ view angle for fabricated lossy medium-based optical cavity using optical images. Reproduced with permission of [43]. Copyright John Wiley & Sons, 2014.

Likewise, for the positional dependence the center wavelength showed 10% variation. The results were further improved by using titanium oxide (TiO${}_{2})$ as a dielectric layer with a phase compensating dielectric overlay; increasing the angle insensitivity up to $70^{\circ }$. The design was modified further by using an overlay on the top of the stack [12] (Figure 2A), which allowed dual mode color filtering as well as phase compensation. The thickness of the TiO${}_{2}$ cavity allowed the sweeping of resonant frequency across the visible regime. Moreover, the design was flexible enough to change color saturation by varying the thickness of one of the Ag mirrors, with a slight change in transmission efficiency.

Zhangyang Li et al. [39] exhibited the performance of ultra-thin, large area, lithography-free metallic films as super absorbers and additive color filters. The design comprised of an insulating film (SiO${}_{2}$) sandwiched between two metallic films (Ag). The design behaved as a super absorber when used in reflection mode with an optically thick layer at the transmission end, hence blocking all the transmission through the device. The transmission and absorbance spectra were studied by varying the thickness of dielectric, i.e., the Fabry-Perot cavity. The resonance wavelength swept across the visible and near infrared regions. The plasmonic effect was not noticed and the power absorption was a maximum at the metal-dielectric interface. In color filters the width of the metallic layers reduced, and the device performance is examined by changing the width of the layer opposite to the light impinging layer. The leaking mode from the cavity was observed due to the small width of the layer at the transmitting end. Furthermore, the dependence of color filters on the angle of incidence and modes of polarization of light source are also taken care of. It was observed that the device was more sensitive to p polarized than s polarized mode and showed angle invariance up to $60^{\circ }$. The results showed 97% of absorption intensity for super absorbers with Full Width Half Maximum (FWHM) of 8 nm. Likewise, transmission color filters achieved over 60% transmission across the visible region. Although the aforementioned examples have exhibited outstanding results, they suffer from high losses due to the presence of metal [12,40]. Hence, CMOS-compatible dielectric materials with a high refractive index [41] can be helpful in reducing losses and low angle variance.

An ultra-thin design (i.e., less than a quarter wavelength of the light) was proposed where an asymmetric FP cavity implies a highly absorbing germanium (Ge) on an optically thick gold (Au) substrate [44]. The obtained results showed maximum absorbance (1−R) due to minimum transmission into the substrate.

Since Ge has high loss and low refractive index when compared with Si, especially its amorphous phase (a-Si) is preferred over it. Moreover, a high-refractive index allows angle invariance, therefore research is inclined towards the material with high refractive indices. An angle robust reflective spectrum filter with high color purity and $\pm 65^{\circ }$ angle invariance is demonstrated by exploiting strong resonance effects in a FP optical cavity containing lossy media [43] (Figure 2C). It acquires remarkably improved colour saturation by choosing a-Si, which has a lower complex dielectric constant. Due to its low absorption and high reflectivity, Ag was used as its metallic layer. The thinner metallic layer at the top (18 nm) allowed the incident light to pass through the structure and simultaneously provide an enhanced reflection from the resonance cavity; however, the bottom Ag is optically dense to block the transmitted light. An organic layer, made from perylene tetracarboxylic bis-benzimidazole (PTCBI), is used to confirm an even thin Ag film that minimizes the scattering.

A CMOS-compatible ultra-thin color filter containing a-Si deposited on an optically opaque thin aluminium film was demonstrated as a subtractive color filter [45]. The reported angle insensitivity is up to 60%. Similarly, a nano-resonantor comprising a-Si between two Ag layers showed angle insensitivity up to $65^{\circ }$ and low FWHM value of approximately 74 nm in reflection mode [43,46]. The wide angle flexible trans transmissive structural colors with high saturation and efficiency were designed by exploiting multi-cavity resonances [46]. The design consists of a stack of alternating Zinc sulphide (ZnS) as a dielectric layer and thin metal (Ag) films. The high refractive index of the dielectric layer allowed $70^{\circ }$ of angle invariance. The design was further coated with a thin ZnS layer as an Anti-reflection (AR) from both the ends (i.e., top and bottom), which further enhanced the color efficiency. The device fabrication used simple e-beam evaporation for layer deposition. In order to avoid precise deposition of the thin metallic layer and AR coating the design was further improved by employing a multi-layer semi-transparent structure for suppressing higher order FP-resonances [47]. The FP design is comprised of a transparent ZnS sandwiched between a thin Ag metallic layer and has an overlay at the top for phase compensation. The cavity is designed to have 3rd order FP-resonance, which is useful to produce good color quality. However, a 5th order resonance is suppressed by employing a thin lossy material layer (Ge) in the center of the ZnS cavity. The angle invariance of $60^{\circ }$ was reported with an FWHM value as low as 36 nm.

A near-unity absorption as high as 99.58% is reported by using chromium (Cr) as a metallic mirror and SiO${}_{2}$ as a dielectric layer [48]. The results showed $\pm 60^{\circ }$ angle tolerance with polarization independence. Similarly, an angle insensitivity of up to $50^{\circ }$ was achieved [49] in reflection color filtering with simple multilayer film deposition. It comprised of lossy a-Si material as a dielectric sandwiched by a thin Cr film and a thick Ag layer. The Ag at the bottom of the stack allows lowest material absorption and highest reflectivity in the visible regime. Likewise, Cr is used as a partial reflection mirror and the absorptive layer. The upper TiO${}_{2}$ layer is used as an effective anti-reflection layer to chrome film, and hence helps in improving the color saturation.

An asymmetric FP-based reflective color filter was demonstrated by [50]. It used lossless metal nickel (Ni) thin film as an anti-reflection coating on the top of the cavity with a thick Al layer at the base which allowed zero transmission. The dielectric consisted of lossless material SiO${}_{2}$. The width of the spacer between Ni and Al allowed color absorption or reflection across the visible regime. The reported color sensitivity invariance is up to $60^{\circ }$ with spatial resolution up to 50,000 dpi. Although [49,50] showed promising results, managing thin lossy metals requires special treatment. Recently, a broadband light absorber was constructed with absorption >95% in the visible region by utilizing thermally evaporated Ag and Au layer [42] (Figure 2B), which exhibit refractive indices much different from the bulk-state value [51]. Nevertheless, the angle invariance was high, i.e., up to $30^{\circ }$.

The summary of all the reported work in terms of FP-cavity performance is given in Table 1.

## 3. Plasmons

A plasmon is a quantum of plasma oscillation. The emergence of plasmons dates back to the Roman Empire’s 4th century AD; the Lycurgus Cup [52,53] is one of the famous examples of plasmons. It contains gold nanoparticles on glass, and filters different (colors) wavelengths in reflection (green) and transmission (red) mode.

Since their inception, plasmons have been implemented for a number of applications like light emitting diodes (LED) [54,55], biosensors [56,57], laser ablation [58,59] solar cells [60,61,62,63], nanolasers [64,65,66], Surface Enhanced Raman Spectroscopy (SERS) [67,68], waveguides [69,70,71] and lithography [72,73].

### 3.1. Surface Plasmon Polaritons

Surface plasmons [74,75,76,77,78,79,80] are an oscillation of electron density with respect to fixed ions localized at surfaces of metallic structures. The induction of surface charge is accompanied with electromagnetic oscillations and is known as Surface Plasmon Polaritons (SPP), where polaritons refer to the coupled oscillation of charged particles and electromagnetic waves. Likewise, SPP are capable of travelling along the interface of two materials having negative and positive permittivity materials, i.e., metal-dielectric interface. Moreover, Surface Plasmon Resonance (SPR) is the resonant excitation of conduction electrons stimulated via incident electromagnetic waves at the metal-dielectric interface.

**Figure 3:**
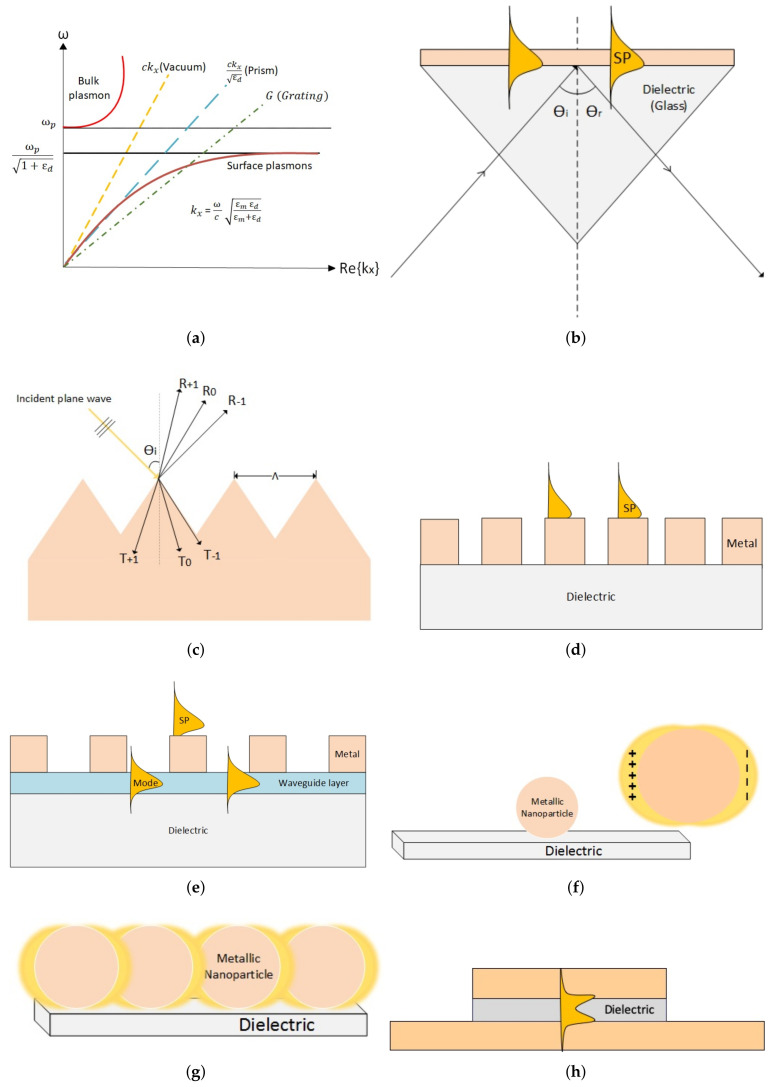
Generating Plasmons. (**a**) Dispersion relation of surface plasmons compared to light in vacuum and in the dielectric medium. Incident light on a (**b**) glass prism with gold sputtered on one of its faces, SPP propagates along the glass and Au interface (**c**) Diffraction grating with period Λ (**d**) shows diffraction grating with varying refractive index and path for the incident light (**e**) represent a GMR-based grating (**f**) The reaction of single nano-particle on dielectric to the incident light (**g**) shows the reaction of lattice of nanoparticles to the incident angle and (**h**) GSP resonance, a special kind of FP-cavity with thin dielectric film and one metallic truncated film on the top.

Figure 3 comprises of various ways of generating plasmons. As shown in Figure 3a, SPRs generated at dielectric-metal interface do not have enough momentum (compared to light) to propagate along the interface. Hence different ways are adopted to fulfill momentum matching needs, such as prisms (Figure 3b), also referred as Otto configuration [76,77] and metallic gratings with period Λ, which reflects and transmits light in different diffracting modes as shown in Figure 3c [76,81]. Similarly, from Figure 3d, by varying refractive indices and path for incident light and by introducing a waveguide between the periodic grating and substrate, which leads to Guided Mode Resonances (GMR) shown in Figure 3e. Furthermore, the localization of SPRs is achieved when light impinges on Metallic Nanoparticle (MNP) on a dielectric substrate as shown in Figure 3f. Moreover, the results can be enhanced by increasing the number of MNP in form of a lattice, which results in coupling all the Localized Surface Plasmons (LSP) due to each MNP in the lattice. The effect of increase in number of MNPs is demonstrated in Figure 3g. Lastly, Gap Surface Plasmon (GSP) can be generated in the dielectric gap between a truncated and extended metallic layers as shown in Figure 3h.

In the subsequent sections, the physics behind different generating mechanism of plasmons along with its notable reported works are discussed in details.

#### 3.1.1. Otto Configuration

Figure 3b shows a basic setup of Otto configuration, where a prism consists of a gold (Au) layer on one of its rectangular faces. When a coherent light wave impinges at the glass/metal (usually Au) interface at an angle $\theta_{i}$, greater than the critical angle $\theta_{c}\left(\arcsin\left(\sqrt{\frac{\epsilon_{m}}{\epsilon_{d}}}\right)\right)$, here $\epsilon_{d}$ and $\epsilon_{m}$ are dielectric constants of glass and metal respectively, and satisfy $\epsilon_{d}>\epsilon_{m}$, and the light is totally reflected back into the glass. However, the electric field propagating along x-direction (TM mode) with amplitude decaying exponentially away from interface represents an evanescent wave. Likewise, the electric field associated with SPPs is given as:

(5) $$E_{j}=\left(E_{x}^{j},0,E_{z}^{j}\right)exp\left\{i\left(k_{spp}x-\omega t\right)\right\}exp\left(-\alpha_{j}\left|z\right|\right);$$

where, *j* is *m* for metal and *d* for dielectric respectively. It is to be noted that the SPP can only be excited with p polarized light, i.e., transverse magnetic (TM) mode propagating along the metal/dielectric interface with amplitude decaying on both sides [82]. The SPP dispersion formula is given as:

(6) $$k_{SPP}=\frac{\omega }{c}\sqrt{\frac{\epsilon \left(\omega \right)\epsilon_{m}}{\epsilon \left(\omega \right)+\epsilon_{m}}};$$

where, ϵ(ω) is the dielectric function of metal and $\alpha_{j}$ in Equation (6) is given as:

(7) $$\alpha_{d}=\frac{\omega }{c}\frac{\epsilon_{m}}{\sqrt{\epsilon \left(\omega \right)+\epsilon_{m}}},$$

and

(8) $$\alpha_{m}=\frac{\omega }{c}\frac{\epsilon (\omega )}{\sqrt{\epsilon \left(\omega \right)+\epsilon_{m}}}.$$

It is to be noted here that Equation (6) is valid for the negative real part and the negligible imaginary part of ϵ(ω). Hence metals like Au, Ag, etc. are suitable for generating SPPs in the visible region.

Although the usage of a prism for generating SPRs at the metal-dielectric interface is an obstacle in its miniaturization, it is used in biosensing devices due to its simple design and sensitivity to change in the refractive index caused by adsorption or desorption of molecules on its surface. On the other hand, grating-based SPRs have been extensively explored due to its miniaturization feasibility and wide range of applications like biosensing [83,84,85,86], solar cells [87], spectral imaging [88] and color filtering [89].

#### 3.1.2. 1D Diffraction Grating

Grating-based SPRs allow coupling of incident light with SPP through grating, where resonance conditions are met through diffraction of incident light.

When a plane wave of monochromatic light of wavelength λ incident at an angle $\theta_{i}$ on a periodically corrugated material of periodicity Λ, the light is diffracted in forward (transmission) and backward (reflection) directions as shown in Figure 3c. The dispersion relation for grating coupling to overcome the momentum mismatch between the energy of impinging photon and of generated electromagnetic waves is given as:

(9) $$k_{SPP}=\frac{\omega }{c}{\sqrt{\epsilon }}_{m}sin\theta i\pm mG;$$

where, *m* is the diffraction order and $G=\frac{2\pi }{\Lambda }$ is the reciprocal lattice with period Λ.

The 1D diffraction grating along a waveguide slab (a slab of material between two materials with lower refractive indices form a slab waveguide) is known as a waveguide mode coupling. The resonance conditions are met when the angle of diffracted mode of incident light matches the angle of guided mode (Figure 3e). These wave are leaked through the gratings and the interference of leaky waves and diffracted wave produces a filter response as shown in Figure 3e. Mathematically, the guided mode resonance condition is satisfied by the dispersion relation of the waveguide coupler and is given as [82]:

(10) $$m\pi =H_{c}\sqrt{\left(k_{0}^{2}\times n_{h}^{2}\right)-\beta^{2}}-\tan^{-1}{\left(\frac{n_{h}}{n_{c1}}\right)}^{2\rho }\times \sqrt{\frac{\beta^{2}-k_{0}^{2}\times n_{c1}^{2}}{\left(k_{0}^{2}\times n_{h}^{2}\right)-\beta^{2}}}-\tan^{-1}{\left(\frac{n_{h}}{n_{c2}}\right)}^{2\rho }\times \sqrt{\frac{\beta^{2}-k_{0}^{2}\times n_{c2}^{2}}{\left(k_{0}^{2}\times n_{h}^{2}\right)-\beta^{2}}};$$

where, $H_{c}$ is the core thickness; β is the propagation constant; $n_{h}$, $n_{c1}$ and $n_{c2}$ are the refractive indices of core, upper cladding and substrate, respectively; and $k_{0}$ is the wave number in free space. Furthermore, ρ is 1 and 0 for TM and TE mode respectively. The guided mode resonance occurs at the phase matching between grating vector and the propagation constant for the guided mode of the waveguide [90]. There are numerous reported designs where the waveguide coupler is exploited for color filtration. Several of the outstanding ones are discussed in this section.

Kaplan et al. demonstrated a highly efficient waveguide coupler based color filter with tunable transmission bandwidth [91]. It consisted of a waveguide layer (silicon nitride) between low refractive indices layer, i.e., silicon dioxide (SiO${}_{2}$). The metallic grating was implemented on the buffer layer at stack (buffer layer/waveguide layer/substrate) using Nano Imprint Lithography (NIL) [72,92].

The impinging source from the grating end was set at TM polarization with zero incidence angle. The wavelength of the transmitted light was dependent on the grating period, while the bandwidth could be adjusted by changing the buffer thickness, hence allowing color tunability by changing the physical dimension of the device. The results were collected through experiments using two different devices: one for red and the other for blue color generation. The experimental results were in good agreement with the simulated results. Although the obtained results showed transmission as high as 90%, with tunable bandwidth, it was highly angle sensitive.

Similarly, an ultra-thin metal (Al)—dielectric (TiO${}_{2}$) GMR structure was studied [93]. An optical thin layer of Al was patterned using e-beam lithography as a cladding on a dielectric guiding layer. As a result, color filtration became possible due to the emergence of the incidence wave and the leaky guided modes of the TiO${}_{2}$ layer. The results showed bandwidth 60, 70 and 90 nm for blue, green and red respectively, with transmission efficiency up to 73%, but with angle variance. In another study, the impact of core material at a low refractive index contrast was implemented using Al${}_{2}$O${}_{3}$ as a core with ultra-thin Al grating [94]. This contrast allowed SPR to occur at Rayleigh anomaly wavelength [95]. The results were quite promising with 80% efficiency and approximately 20 nm bandwidth. The device was fabricated using a regular lift off process with the help of laser interference lithography. However, the design exhibited angle sensitivity.

On the other hand, the angle sensitivity of 1D grating was made useful in a reflection-based dielectric-based guided mode resonant color filter design with angle tunability for additive color filters. It showed efficiencies of approximately 94%, 96% and 99% with green and red color filters, respectively. The designed silicon nitride grating was patterned on a glass substrate using UV-laser interferometric lithography. The reported pixel bandwidth was 12 nm [96]. Similarly, Koirala et al. [97] presented a highly efficient polarization controlled structural color filter based on an ultra-thin 1D resonant Al-based grating structure with silicon nitride as a guided layer. It was proposed that 0.5 duty cycle (required to fulfill the guided mode resonant condition) allows transmission in TE and TM mode.

**Figure 4:**
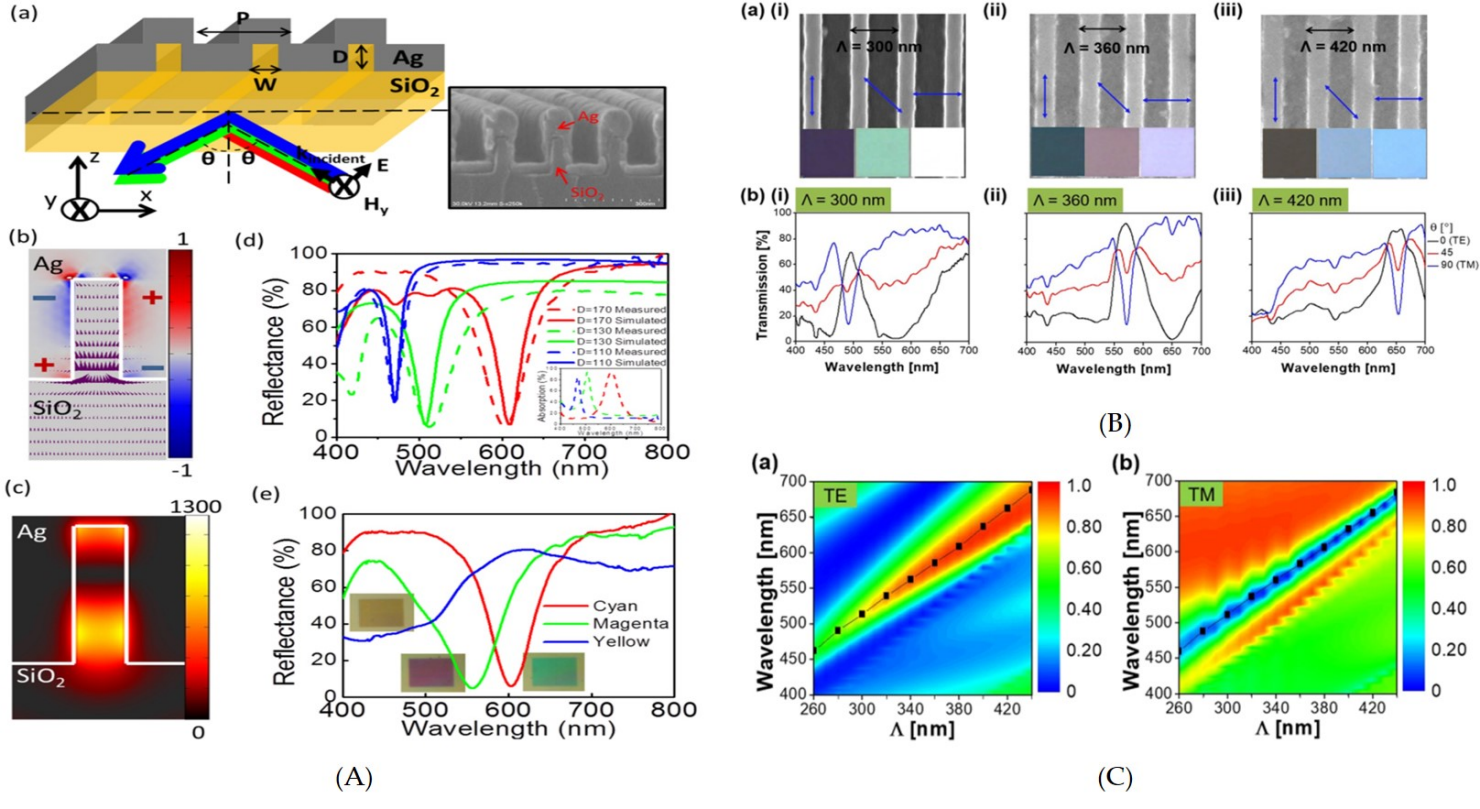
(**A**) Schematic of plasmonic nano-cavity along with its fabricated device is illustrated. The charge distribution and Poynting vector direction is shown with the help of a simulator. The impact of dimension of nanoslits on reflection spectra is further shown theoretically and experimentally and results for CMY colors are produced [100]. (**B**) Scanning Electron Microscope (SEM) images of fabricated multicolor pixel and their spectral transmission. Camera image of vivid color produced shows its dependency on the grating period and angle of polarization (**C**) Verification of GMR for TE and TM mode transmission spectra [98].

This is, as per our best knowledge, the first time where such a setup was used in transmission mode. The same design was exploited by replacing Al gratings with hydrogenated amorphous silicon (H:a-Si) grating in an all dielectric polarization-tailored trans-reflective structural multicolor pixels design [98]. It showed 95% and 90% transmission efficiency in TM and TE mode, respectively (Figure 4A,C); however, the angle tolerance improved to $35^{\circ }$. Similar results were achieved by Al sub-wavelength grating with slits filled with dielectric (SiO${}_{2}$) allowing enhanced EOT for TE mode [99] by exploiting GMR, hence the SPRs are responsible for EOT in TM mode. The gratings are further covered with a thin overlay of SiO${}_{2}$, which improved the device operation. Although color intensity improved in TE, the increase in bandwidth resulted in low color saturation. Likewise, the device remained angle sensitive.

A highly transmissive plasmonic subtractive color filter was proposed in [101]. The desirable results were achieved by exploiting the counter-intuitive phenomenon of Extraordinary Low Transmission (ELT) [102]. An optically thin silver (Ag) film patterned in 1D nanogratings was fabricated to achieve subtractive color. The transmission valley, with 60 to 70% efficiency, can be tuned across the visible regime by varying the periodicity of the silver gratings. In addition, this design is capable of filtering the subtractive color with only a few nanoslits, close to the optical diffraction limit (λ/2, i.e., 200 to 350 nm).

The dependence of GMR based color filters on light incidence angle variance and direction of polarization has been a matter of concern [91,103,104,105,106]. A symmetric two-dimensional grating pattern, 2D version of [91], resolved the issues faced in the aforementioned examples. A polarization independent bandpass filtering was made possible by the GMR between orthogonally diffractive waves and both TE and TM mode [90]. The change in grating peak resulted in a change in center frequency. The reported design exhibited arrow bandwidth of 13, 14 and 17 nm for blue, green and red color. However, the efficiency remains constant at 85%. Moreover, the fabrication steps included e-beam lithography and dry etching. Likewise the 2D version of [107] generated a wide polarization controlled color gamut [108].

Ryan Wuo et al. [100] numerically and experimentally demonstrated localized resonance in silver (Ag) nanoslits by light funneling (Figure 4A). Here the silver was conformally deposited on the silica grating as shown. The design of the grating with certain period, width and periodicity concentrated incident light into silica nano-grooves, which behave more like nano-resonators. When the light is impinged on the device from the silica end, it induces charge polarization inside the grooves. This charge induction changes the E-field of incident light, hence trapping it in the grooves. The resonant wavelength is tuned across the visible regime by changing the width and depth of Metal Insulator Metal Fabry-Perot (MIMFP) nano-grooves.

The 1D diffraction-based color filters allow an undesirable angle sensitivity in the results. Although fabrication of angle nano-resonators requires more fabrication steps and advanced technology, it happens to be a better option in terms of its performance, e.g., the polarization angle and angle insensitivity.

Recently, diffraction gratings have been studied as wave retarders [107]—capable of changing the direction of light polarization. A 4-fold color filtering was achieved in the visible region by utilizing Plasmonic Phase Retarder (PPR). It consisted of a periodic array of silver nanowires, which supported Localized Surface Plasmon Resonances (LSPRs) that can induce strong phase shift for TM or TE mode [109]. Hence, with the help of a colorless analysing polarizer, it produced four different colors from one fabricated device.

The summary of the discussed examples is given in Table 2, where all the designs are polarization dependable.

### 3.2. Localized Surface Plasmonic Resonances

Localized Surface Plasmon Resonance (LSPR) involves the combined oscillation of MNP and the associated oscillation of the electromagnetic field (Figure 3f). The resonance wavelength $\left(\lambda_{res}\right)$ depends on the shape, size, composition of MNPs, and the local optical environment of the particles. It generally occurs in the visible to near IR spectra for NP of noble metals (Au, Cu, and Ag). Mathematically, dependence of extinction optical $\left(\sigma_{ext}\right)$ on shape, density, dimensions and surrounding environment of a spherical MNP is given as [110,111]:

(11) $$\sigma_{ext}\left(\lambda \right)=\frac{24\pi^{2}N_{A}^{3}{\epsilon_{out}}^{\frac{3}{2}}a^{3}}{\lambda \ln\left(10\right)}\left[\frac{\epsilon_{i}\left(\lambda \right)}{{\left(\epsilon_{r}\left(\lambda \right)+\chi \epsilon_{out}\right)}^{2}+{\epsilon_{i}}^{2}}\right];$$

where, $N_{A}$ is the areal density; *a* is the radius; and $\epsilon_{out}$ is the dielectric constant of the surrounding medium of the nano-structure. Here, $\epsilon_{m}$ is assumed to be the positive real integer and is independent of λ. It is to be noted that $\epsilon_{i}$ and $\epsilon_{r}$ are the imaginary and real components of the metal dielectric function respectively. Additionally, χ gives the aspect ratio of the nanostructure.

The spectral bandwidth of Localized Surface Plasmon (LSP) is around 80∼100 nm FWHM, which is far greater than the spectral bandwidth of the change in the reflectance dip of SPPs, i.e., 50 nm. This increased spectral width can be reduced by increasing the number of nanoparticles.

The induced dipole moment p due to electromagnetic field (*E*) in a single metallic nanoparticle is given as

(12) $$p=\alpha E_{0};$$

where, α is the polarizability and can be obtained using a quasistatic approach. By considering the size of particles smaller than the light involved polarizability can be derived from Equation (12) as per the following:

(13) $$\alpha =4\pi a^{3}\frac{\epsilon (\omega )-1}{\epsilon (\omega )+2};$$

where, *a* is the radius of nanoparticle, ϵ(ω) is the dielectric constant, and α is the measure of effect of applied electric field on the particle. Hence, α is directly proportional to charge displacement within the particle. The resonance occurs due to restoring Coulomb force inside the particles by satisfying ϵ(ω)+2=0 or ϵ(ω)=−2. Here the strength of the force determines the resonance frequency while the damping determines resonance width [112,113,114,115,116,117].

William L Barnes [118] elucidated the effect of the shape of nanoparticles on the resonance frequency in terms of polarizability. As per the author’s mathematical and theoretical explanation polarizability depend on the composition of the particle as well as the environment. In addition, it also depends on the net electric field inside the particle.

The quality factor of the resonance is the ratio of resonance wavelength and the width of resonance and it has the highest value when Im(ϵ(ω)+2)=0. Since the dielectric medium of metal is a complex value, therefore the denominator of Equation (13) cannot reduce to zero. Later, Wang and Shen [89] proved with their quasi-static approximation that the quality factor actually depends on the dielectric medium of the particle. Hence LSP has been a disappointment for different application where a high Q factor is desired.

A significant improvement in Q factor arises when two or more than two nanoparticles are brought close together. The interaction of the EM fields between nanoparticles results in coupled SP modes [119] and field enhancement. Similarly, metallic nano-particles arranged in a lattice experience surface lattice resonance (SLR) due to the coupling of LSP (Figure 3). It leads to significant narrowing of the spectral bandwidth and results in high Q factor [120]. The position of resonance in a lattice depends on the shape, size, incidence angle and the refractive index of the metal-dielectric layer [121,122,123,124].

The emergence of Extraordinary Optical Transmission (EOT) [9] due to coupling of surface plasmons in a surface lattice has been a breakthrough. It has been widely used and its applications include drug delivery [125], imaging [126,127], color filtering [128,129], high resolution display [130] and high sensitive sensing [131,132]. Since the research in the field of plasmonics is expedited with the discovery of new materials and design fabrication techniques [72,92,133], this review focuses on and evaluates those developments. The periodic subwavelength nano-particles of any shape, material, and sufficient number for LSPR, behaves in the same manner to incident light as electricity to atomic crystals at the subatomic level. In this section, the reported work to date for photonic crystals is discussed and evaluated in terms of its performance as a color filter. The section includes nano-porous films, hybrid structures, metasurfaces and GSP-based designs studied so far.

#### 3.2.1. Nanoholes

A porous metallic film on dielectric when excited by a white light source results in EOT and only allows a light of certain wavelength $\left(\lambda_{res}\right)$ to pass through it. The $\lambda_{res}$ depends on the periodicity and geometry dielectric constant of metal and dielectric respectively, and a lattice arrangement of holes [134,135,136,137,138,139,140]. Similarly, the mode of operation, i.e., transmission [141] or reflection [142] of the structure, also depends on the shape of the nanoparticles.

The localized field in nanoholes and nanoparticles is widely investigated [143]. It has been reported that LSPR in nanoholes is dependent on the lattice periodicity; however, it is less affected in a metasurfaces array. Similarly, the fabrication process of nanoholes on metal films is easier as compared to metasurfaces. Nevertheless, an increase in resonance quality in nanoholes is quite challenging [112,144,145,146,147,148]. Therefore, the application of a perforated metal film (usually bio-compatible, i.e., Au/Ag) on the dielectric layer is limited to biosensing sensors [149,150,151,152,153,154,155,156,157]. On the other hand, the application of subwavelength nanoholes is not limited to electromagnetic waves, it has been theoretically proven that various acoustic absorbers, under coherent conditions can be attained using the effective deep-subwavelength holey rigid plate, such as steel [158].

A dielectric-based 2D subwavelength dielectric grating was proposed for Back Side Illuminating (BIS) CMOS Image Sensor (CIS) technologies [159]. It consisted of a hexagonal nanohole array for red and blue color, and a square nanohole array for green color production with a silicon spacer between photodiode layer and grating. The device was fabricated with poly-Si materials prepared by a-Si deposition followed by a high-temperature furnace annealing method [160,161]. The design exhibited promising transmitted results, i.e., 60–80% efficiency with angle invariance up to ±20%. The high transmission efficiency was achieved due to the high refractive index and low absorption of a-Si which helped in enhancing the spectral features [162]. Besides, holes of different shapes have also been explored, and have shown promising results, and has provided a flexible tuning mechanism. For instance, a CMOS-compatible polarization-switchable array of asymmetric cross-shaped nanoholes in an Al ultra-thin film was reported [163]. The mechanical-based optical filtering enabled a single design to encode two information states, which could be decoded by changing the polarization angle of incident white light. However, the maximum transmission efficiency of subtractive colors was insufficient, i.e., 14% with incident angle variance.

The nanoholes with FP etalon have demonstrated efficient results. Chang et al. [164] demonstrated an efficient structural color printing filter based on plasmonic metasurfaces with a thin MIM stack. In order to avoid color crosstalk, holes on perforated Ag film were milled, through Focus Ion Beam (FIB) [165], in a hexagonal array. The design was further covered with a thin transparent polymer layer, PMMA. The change in $\lambda_{res}$ due to overlay is covered by geometrical adjustments.The design showed promising results and allowed pure perfect absorption resonances of approximately 90% with a high Q factor. Furthermore, the design was angle insensitive up to approximately $70^{\circ }$, accredited to the angle robustness of the FP cavity. However, the usage of Ag in this design had introduced additional fabrication steps like adhesion layer between glass and Ag and PMMA layers, to avoid Ag oxidation. Recently, a transmission efficiency of 60% was exhibited by increasing the density of coaxial holes of optimized size in a coaxial hexagonal lattice (with bigger holes), on a MIM stack of Al/glass/Al [166]. Although the design exhibited angle invariance of approximately 60${}^{\circ }$, the color quality reduced with the increase in number of the coaxial holes.

#### 3.2.2. Nanohole-Nanodisk Hybrid Color Filters

**Figure 5:**
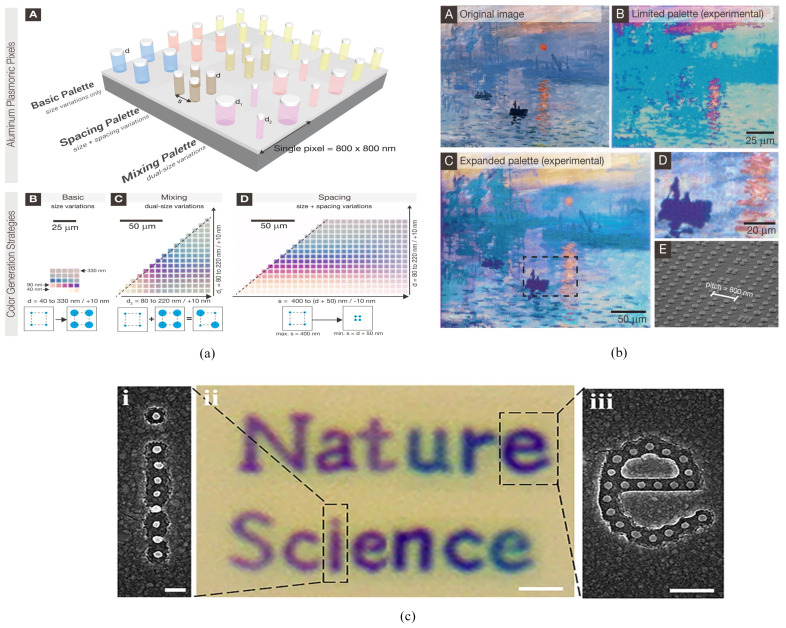
(**a**) schematic diagram of aluminium based nanohole-nanodisk hybrid structure. (**b**) The impact of variation in diameter, periodicity and (spacing) diameter and periodicity on the color image due to color toning and mixing is demonstrated experimentally. Reproduced with permission of [170]. Copyright American Chemical Society, 2014. (**c**) Color printing resolution test pattern for LSPP based nanostructures at the scale bar of (i) 200 nm, (ii) 1 μm and (iii) 500 nm [171].

Kumar et al. [167] with a nanohole-nanodisk hybrid structure, to our best knowledge, was the first one who demonstrated a system that could achieve bright-field color prints with resolution up to diffraction limit (i.e., 10,000 dpi). To obtain desirable results, the scattering strength of resonators (nanoparticles) was increased by raising them above a metal back reflector (Ag/Au), hence the light of certain wavelength was reflected back to the viewer‘s eye. A full palette of colors was obtained by changing the diameter and gaps between the nanodisks without varying the periodicity of the array. The dips in the reflected spectrum showed the presence of Fano resonance [168] that was the outcome of a broad spectrum of nanoholes and nanodisks with sharp peaks of SPRs. This work was further extended by using, aluminium instead of Ag. It paved the way towards a cost-effective and stable structure, as compared to the short shelf life of Ag [169]. Its high-throughput lithography approaches also gives it an edge over Au and Ag. Moreover, the range of printable plasmonic colors was expanded from approximately 15 to more than 300 colors by spatially mixing and adjusting the nano-scale spacing of the aluminium-based discrete nanostructures [170] (Figure 5a,b).

Recently, spatial resolution of approximately 141,000 dpi with a wide gamut and large angle invariant was achieved by implementing circular nanohole-nano-disk hybrid nanostructure arrays based on the uncoupled Localized Surface Plasmon Polaritons (LSPPs) [171] (Figure 5c). The $\lambda_{res}$ spanned across the visible regime by varying the diameter of the nanodisk-nanohole structure rather than its periodicity. Hence a single nanodisk-nanohole could be operated as an individual pixel resulting in sub-diffraction limit resolution. The results included theoretical as well as experimental results for subtractive colors.

An ultra-thin hexagonal nanodisk-nanohole hybrid structure array generated high gamut, high color brightness and polarization independent subtractive colors with 77,000 dpi [172]. Here, the excitation of short range SPR [173] and LSPR contributed to the ELT effect for the designed structure.

A structural design was demonstrated to generate structural color through triangular-latticed square nanohole arrays in Al film with bottom Al nanodisks [174]. The design was fabricated using e-beam lithography and metal deposition by adjusting the period and side length of nanohole arrays, colors, across the entire visible regime, with tunable brightness, hue and saturation, and pixel density of 12,700 dpi. However, the filtered colors were highly saturated.

An angle-insensitive of scratch-resistant hybrid disk-hole plasmonic based color filter was demonstrated by utilizing the PMMA layer on a glass substrate [175]. For fabrication, NIL was employed, where the silicon masters used for embossing were fabricated using EBL and subsequent dry etching. Furthermore, the samples were made using hot embossing and metal evaporation. Later, Al was deposited using electron-beam evaporation. The hybridization of plasmonic modes due to nanodisks and nanohole array offered angle-insensitivity up to $60^{\circ }$.

#### 3.2.3. Metasurfaces

Metamaterials with subwavelength thickness, such as nanodisks, nano spheres, nanotubes and thin films, are capable of manipulating the wave front of impinging light by changing its phase [176], amplitude [177] or angle of polarization [178]. Its applications include: cloaking [179,180], polarization manipulation [181,182,183], high resolution lenses [184,185] and perfect absorbers [186]. The performance of metasurfaces as color filters have shown remarkable results; this section includes the reported work in this field.

**Figure 6:**
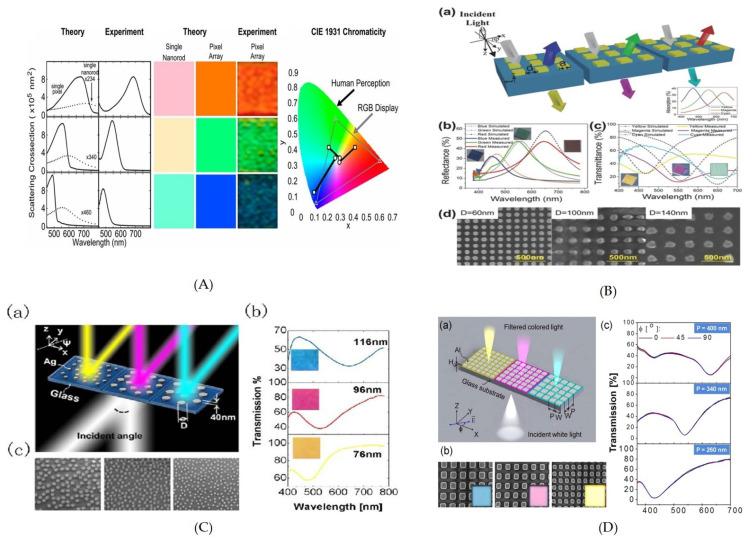
(**A**) Experimental and theoretical results of vivid colors produced due to Al nanorod as a single nanorod and in hexagonal array [189]. (**B**) Schematic diagram of Ag nanopatch on glass substrate with different periodicities allowing angle invariant (up to $60^{\circ }$) additive and subtractive color filtration in transmission and reflection respectively. The plot for simulated and experimental spectra with the color image inset shows the obtained results. SEM gives a close look at the fabricated nanopatch array. Reproduced with permission of [187]. Copyright John Wiley & Sons, 2016. (**C**) schematic diagram of randomly distributed Ag nanodisks, the $\lambda_{res}$ was swept by varying the diameter of the nanodisks. The transmission spectra with color image in inset shows the color quality produced. Reproduced with permission of [190]. Copyright The Optical Society, 2013. (**D**) Schematic diagram of thin Al nanopatch array on a glass substrate, this design filters out white color into distinct subtractive colors by varying the pitch of array. The color quality is evident in SEM and camera image of the fabricated filters. The polarization independence is verified by plotting reflection spectra at different polarization angles. Reproduced with permission of [191]. Copyright American Chemical Society, 2014.

An ultra-thin Ag patch array on silica was demonstrated [187] (Figure 6B). The Ag was deposited using magnetron sputtering followed by FIB. The $\lambda_{res}$ is affected by change in patch dimensions, hence it can be swept across the visible regime. The structure showed the angle sensitivity up to $60^{\circ }$ in transmission and reflection modes. The angle insensitivity is credited to LSPR at nano patch array. However, the transmission efficiency is up to 40%, and usage of Ag for CMOS devices has its own disadvantages [188].

A 2D array of opaque, but physically thin Al nanopatch array on glass, demonstrated highly efficient plasmonic subtractive color filtering by capitalizing on SP-mediated selective transmission through it. Here, the transmission was suppressed by opaque nano-patches instead of ELT. The spectral position of the transmission valley was governed by varying the periodicity of the nanopatches, and realized a palette of subtractive colors [191] (Figure 6D).

A highly polarized angle-sensitive CMOS-compatible aluminium nanorods arranged in a hexagonal lattice and excited via a prism produced vivid, high-contrast colors of 100:1 with the footprint of 25 μm × 25 μm (Figure 6A). Here the far-field diffractive coupling is exploited to narrow down the bandwidth down [189,192], and the physical dimensions of nanorod allowed additive color tuning across the spectrum. However, the usage of a prism for excitation is a hurdle in its integration and miniaturization.

An array of two dimensional randomly distributed silver nanodisks on a glass substrate with improved angle insensitivity up to $60^{\circ }$ for reflected color filters was demonstrated [190] (Figure 6C). It was possible by defying hybridized LSPR and isolating the nano-disks. The uncoupled LSPR allowed better angle sensitivity as compared to the coupled LSPR. The color was tuned by varying the diameter of the nanodisks, while the minimum distance, to avoid LSPR coupling, between nanodisks was kept up to 80 nm. A broad series of color filters with colors varying from yellow to cyan shown were fabricated with the nano-disk diameter ranging from 66–166 nm. The circular shape of the nanostructure allowed polarization insensitivity and showed good brightness and high color contrast across the visible region. The angle insensitivity was further improved to $70^{\circ }$ with randomly distributed nanodisks and nanoholes fabricated with hydrogen silsesquioxane (HSQ) and Ag films on silicon substrate [193]. The LSPR based color filters are further summarized in Table 3.

#### 3.2.4. Gap Surface Plasmons

A Gap Surface Plasmon (GSP) is a special kind of plasmon metasurface, where a subwavelength dielectric spacer is sandwiched between an optical thick metal and metallic subwavelength particles arranged in a periodic or quasi-periodic arrangement [196]. The subwavelength-thin dielectric spacer enhances the field in MIM and allows near-field coupling between the SPP of the two interfaces [197] (Figure 3h).

The FP resonance condition for MIM with truncated upper metallic layer of width *w* is given as [198,199]:

(14) $$w\frac{2\pi }{\lambda }n_{GSP}=m\pi -\phi .$$

Here, $n_{GSP}$ is the effective refractive index of GSP, λ is the vacuum wavelength, ϕ is the unknown phase change due to reflection at the nanodisk boundary and m is a positive integer account for resonance mode. The applications of GSP resonators include: flat lenses [200,201], surface wave couplers [202,203], holograms [204,205], beam-steerers [206,207] absorbers [208,209], polarization controllers [210,211], and dynamically reconfigurable metasurfaces [206,212]. As per the focus of this review, the reported work on GSPR-based color filters is discussed and reviewed in this section.

**Figure 7:**
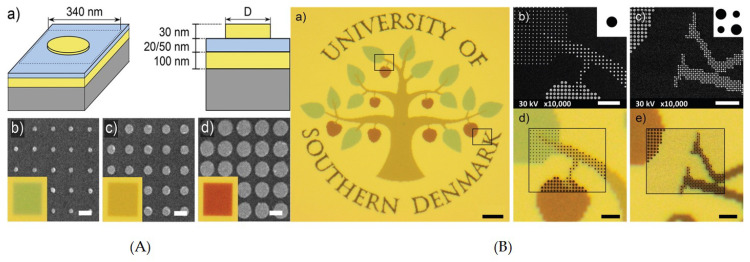
(**A**) Schematic diagram of a circular gap plasmonic nano microscopic images of the highly uniformed colors due to an array (**B**) Precise color printing on an optical microscopic image demonstrate bright colors with high contrast, and demonstrate that even single pixel details are colored and discernible. Reproduced with permission of [194]. Copyright American Chemical Society, 2014.

A plasmonic color filter with subwavelength resolution based on MIM configuration capable of supporting GSP was demonstrated [194] to effectively reflect different colors at subwavelength scale (Figure 7). Here an array of 340 nm period containing circular gap-plasmon resonators of Au/SiO${}_{2}$ and Au was fabricated with single-step electron beam lithography. Here $\lambda_{res}$ is varied by varying the diameter of the nanodisks and allows angle insensitivity up to $60^{\circ }$ with polarization dependence. Furthermore, the $\lambda_{res}$ is immune to the presence of overlay, hence it allows chemical and mechanical stability to the device.

The performance of the aforementioned example was further improved by utilizing $Al/Al_{2}O_{3}/Al$ MIM stack in nanodisk GSP resonantors [213]. Here each nano-resonantor is treated as an individual color element. Hence, the resultant color obtained is the effect of color absorption due to each of the resonantors in an array. The change in the nanodisk diameter and array periodicity covered the entire visible regime with saturated colors and dark colors at the optical diffraction limit. The presence of Al makes it robust and reliable, discarding the need of overlay, which can be red shift $\lambda_{res}$ in this case.

## 4. Dielectric-Based Color Filter

As per scattering theory, all scattering bodies can be represented by effective magnetic and/or electric polarizability densities. Light scattering by small particles is based upon the Mie solution of the diffraction problems [25]. The scattered field of a single isolated dielectric sphere with radius $r_{o}$ and relative refractive index n can be decomposed into a multipole series with the $2^{m}$-pole term of the scattered electric field proportional to

(15) $$a_{m}=\frac{n\psi_{m}\left(nx\right)\psi_{m}^{\prime }\left(x\right)-\psi_{m}\left(x\right)\psi_{m}^{\prime }\left(nx\right)}{n\psi_{m}\left(nx\right)\xi_{m}^{\prime }\left(x\right)-\xi_{m}\left(x\right)\psi_{m}^{\prime }\left(nx\right)};$$

whereas, the $2^{m}$-pole term of the scattered magnetic field is proportional to

(16) $$b_{m}=\frac{\psi_{m}\left(nx\right)\psi_{m}^{\prime }\left(x\right)-n\psi_{m}\left(x\right)\psi_{m}^{\prime }\left(nx\right)}{\psi_{m}\left(nx\right)\xi_{m}^{\prime }\left(x\right)-n\xi_{m}\left(x\right)\psi_{m}^{\prime }\left(nx\right)};$$

where, $x=k_{o}r_{o}$, and $k_{o}$ is the free-space wave number and $\psi_{m}\left(x\right)$ and $\xi_{m}\left(x\right)$ are Riccati-Bessel functions. The primes indicate derivation with respect to the arguments. The scattering coefficient $a_{m}$ and $b_{m}$ are related to the electric and magnetic response of the sphere, respectively.

Here, the first and second highest wavelength resonances of dielectric spheres correspond to the electric and magnetic dipoles [214]. The dielectric resonators [215,216] are capable of concentrating electromagnetic energy in different modes with the increase in refractive index *n*, which results in higher Q factor and allows sharp Mie resonances in small spherical particles with a large permittivity [217]. Similarly, the magnetic response of dielectric resonators in reaction to the magnetic component of external electromagnetic waves has significance in the implementation of negative index metamaterials (NIM) with isotropic properties [218]. On the other hand, the metallic nanoparticles mostly demonstrate anisotropic optical magnetic response and are prone to significant ohmic losses which aggregate with the increase in frequency, hence making it unsuitable for lower wavelength signals like blue color in the visible regime [219]. Due to a high Q-factor and low loss, dielectric-based color filters with high permittivity material have been studied and demonstrated extensively. This section includes extraordinary research work done in this field.

**Figure 8:**
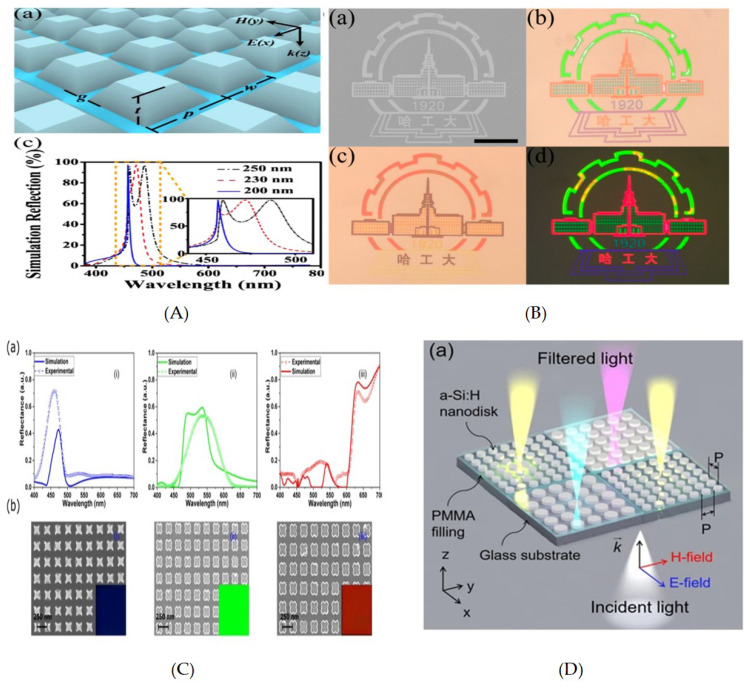
(**A**) Schematic diagram of TiO${}_{2}$ based color filter, the reflection spectra shows dependence of filter on the device dimensions (here the width of the bottom face w is varied from 200 to 250 nm). (**B**) Color image printing is exhibited with the top view SEM image of the logo with transmission and reflection colorful images under bright field microscope. Reproduced with permission of [220]. Copyright American Chemical Society, 2017. (**C**) Simulated and experimental results for additive colors of all dielectric metasurfaces based on cross-shaped resonators. Reproduced with permission of [221]. Copyright American Chemical Society, 2017. (**D**) Schematic diagram of 2D lattice of a-Si:H nanodisks on a glass substrate [222].

A full color printing with titanium oxide (TiO${}_{2}$) metasurfaces with trapezoid shaped nanostructures arranged in square lattice was demonstrated [220]. High reflection peaks, along with bright contrast, were obtained across the visible regime. The fabrication involved a typical lift-off process. Taking advantage of the Fano resonances, the reflected color was also achieved from TiO${}_{2}$ metasurface with much smaller pixel sizes. Thus bright, high contrast and high-resolution structure colors were generated (Figure 8A,B).

It has been notified that color saturation for higher desired wavelength is impaired due to the presence of high order Mie resonances (quadruple mode) at lower wavelength [223,224]. This was addressed by exploiting multi dielectric metasurfaces with SiO${}_{2}$, TiO${}_{2}$ and Si${}_{3}$N${}_{4}$ from top to bottom on silica substrate [225]. The index matching between layers allow multi polar modes suppression at non-resonant wavelength and results in extraordinary enhancement in the monochromaticity of reflection spectra. It expanded unprecedentedly and acquired 171% sRGB space, 127% Adobe RGB space and 57% CIE space.

Recently, it was proposed that application of Rayleigh anomalies at relatively short wavelength in Si${}_{3}$N${}_{4}$ (nanopillars) metasurface on quartz substrates results in higher modes suppression. However, the lateral angle of incidence was employed for producing vivid colors in transmission modes [226].

A cross-shaped a-Si based 2D array of nano-resonators demonstrated by Vishal et al. [221] showed polarization insensitive results with high quality and a wide color gamut (Figure 8C). High purity colors were obtained by optimizing resonance properties, easily tunable through change in structural parameters. The obtained resonant wavelength is further tuned by varying the aspect ratio of nano-resonators. The obtained results showed polarization insensitive independent resonances with high transmission efficiency. However, the issue of incidence angle could not be addressed.

All-dielectric polarization dependent reflection color pixels with a subwavelength resolution of up to 85,000 dpi have been demonstrated [223]. Here the c-Si nano block resonators were arranged in a square lattice allowing distinct vivid colors across the visible regime, by varying its physical geometry. The results showed that these individual pixels could be distinguished easily in a chequered pattern, and do not allow color mixing. However, an incidence angle insensitivity of only $20^{\circ }$ was achieved. The subwavelength resolution was further improved to 100,000 dpi by exploiting mono crystalline Si nano-disks resonantors with metal mask on it [227]. This offered high resolution, by weakening the interaction between two resonators to such an extent that even with a pixel containing a single nano-resonator, the filtered color will not be mixed.

A substractive CMY color filter showed high efficiency and color quality with a-Si-Al hybrid nanodisk metasurfaces on Si substrate [228]. The nanodisk supports MD resonance mode, and is confined to the nanodisk due to Al nanodisk at the top. A near-zero reflection dip was reported and swept across the visible regime by changing the diameter of the nanodisks. However, incidence angle invariance of only $25^{\circ }$ was reported. The results for yellow color could further be improved by implementing a rectangular lattice as theoretically presented [229]. Ninety percent efficient reflective color filters with hydrogenated a-Si nanodisks in a square lattice were realized by [222] (Figure 8D). The wavelength-dependent filtering characteristics are governed by ED and MD resonance via Mie Scattering. The vivid subtractive colors were tuned by varying structural parameters like the period and radius of the nanodisks.

All dielectric-colored silicon-based metasurfaces (silicon nanopillars) etched on a glass substrate with the period of 1 μm were theoretically and experimentally studied [230]. The farther apart nanopillars defied the coupling and each nano-resonantor worked as an individual color filter, where the response of ED and MD can be tuned by changing the aspect ratio of each nanoparticle.

Furthermore, an economical solution for broadband anti-reflection through Si surfaces textured by laser-based method has been proposed [231]. The study showed that well-ordered Si Nanowires (SiNWs) with high aspect ratios improves the performance in UV-visible-NIR range. Likewise, decoration of Si with MNPs also reduced the optical reflection. The hybrid anti-reflection surfaces demonstrated reflection below 1%.

All studied Mie Scattering based color filters made of high refractive index nanoresonantors are summarized in Table 4.

## 5. Liquid Crystal-Based Plasmonic Color Filters

The active plasmonic color filters are the ones that can encode more than one color state in it. These devices generally employ active mediums such as liquid crystal [232,233], elastic polymer [234,235], chemical reduction/oxidation [236,237] and molecular machines [238,239]. But liquid crystal, with the highest birefringence on the refractive index, low threshold on transition among different states, and versatile driven methods to create transitions, stands out from all the aforementioned kinds. Moreover, their large birefringence span across the visible regime and near infrared [240], high throughput, compatibility with opto-electronic materials, and flexible control of alignment of liquid crystals, i.e., via light, electricity and acoustic waves, makes it the best candidate.

**Figure 9:**
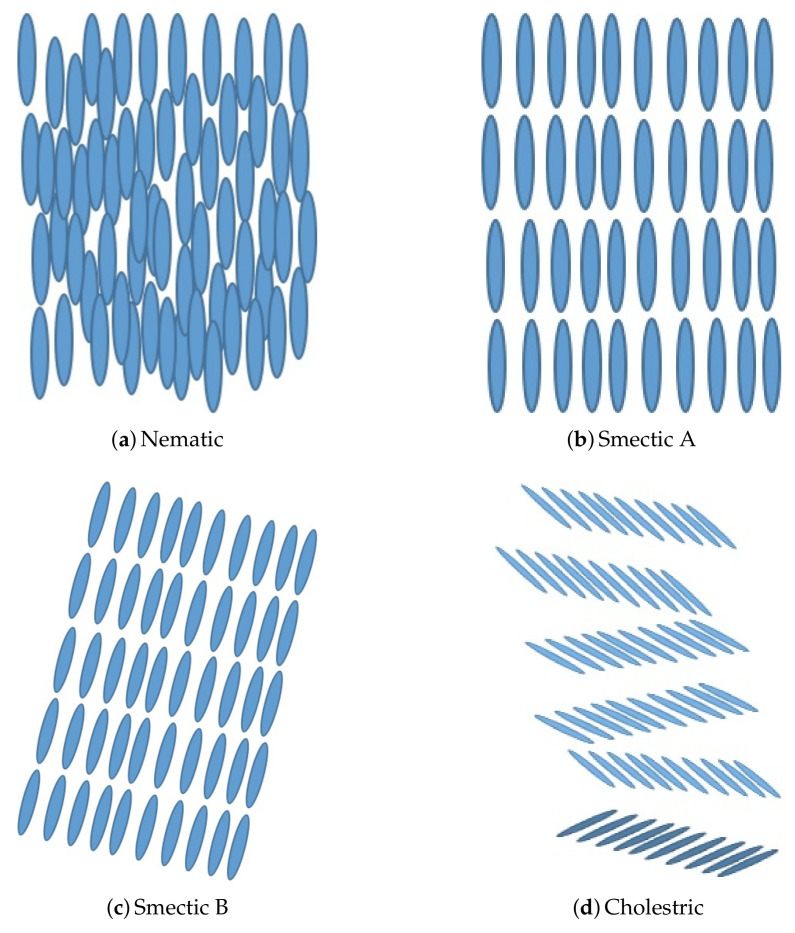
Phases of liquid crystals. (**a**) Nematic phase. (**b**) Smectic A phase. (**c**) Smectic B phase. (**d**) Cholestric phase.

In general, there are three phases of liquid crystals: 1, nematic; 2, smectic; and 3, cholesteric. In the nematic state (Figure 9a) the molecules are obeying the translational order only, while in the smectic phase there is translational as well as orientation order in all the molecules (Figure 9b,c). In the cholesteric phase, a special kind of nematic phase, (Figure 9d) the phase of the liquid shows the distribution of molecules at several planes that are perpendicular to the helical axis. The molecular chirality governs the twist direction i.e., right-handed or left-handed.

The most remarkable characteristic of liquid crystals is their anisotropic refractive index. One refractive index corresponds to light polarized along the director of liquid crystals, while the other is for light polarized perpendicular to the director. Hence, magnetic and electric field components will propagate through the liquid crystal at different speeds, which results either in or out of phase at the exit from the crystal. Furthermore, the polarization state of light will change at the exit due to optical anisotropy. Likewise, the birefringence decreases with the decrease in temperature and ends up at the isotropic phase [241,242].

Since the orientational order and birefringence can be tailored easily via electric, weak magnetic and optical fields, it has been used in numerous applications such as Liquid Crystal Display (LCD) for watches, television, cellphones, spatial light modulators for adaptive optics in real time optical imaging, optical phase array for beam steering, and attenuators for telecommunications [243].

**Figure 10:**
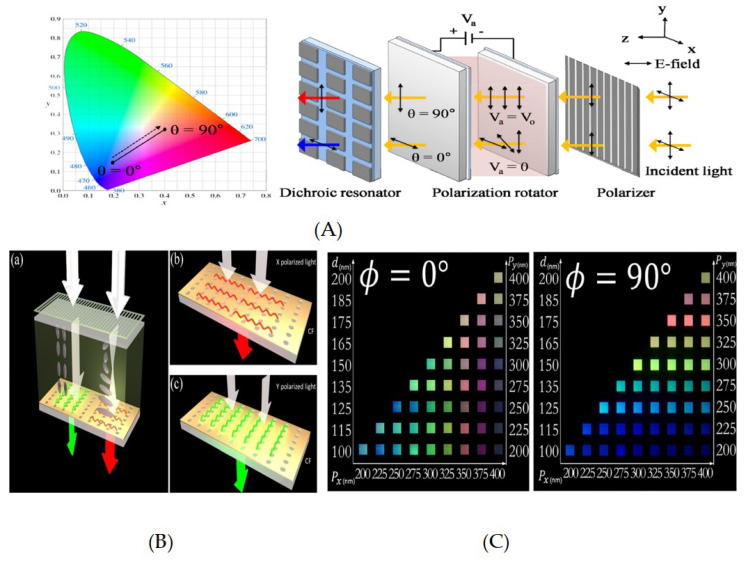
Liquid crystal-based color filters. (**A**) Setup of electrically tunable color filter exploiting a visible dichroic resonator with subwavelength metal-dielectric resonant structure in conjunction with a LC-based polarization controller. Reproduced with permission of [19]. Copyright The Optical Society, 2013. (**B**) Schematic diagram of electrically tunable color filter exploiting TN-LC and rectangular lattice of nanoholes on Al film to control the polarization of incident light, and (**C**) Optical photographs of printed colors in TM and TE mode. Reproduced with permission of [244]. Copyright American Chemical Society, 2017.

In this section recent research examples of liquid crystal plasmonic-based color filters are studied and reviewed. A highly efficient electrically tunable color filter with dichoric resonantors was achieved by exploiting a LC-based polarization rotator [19] (Figure 10A). The variation in applied voltage rotated the polarization in LC and affected the results from the dichoric resonators. The dichoric resonators comprised of guided mode resonance through an $Si_{3}N_{4}$ dielectric layer and the diffraction due to 2D rectangular Al-grating. The LC-based polarization rotator allowed electrical color tuning across the visible regime with high efficiency up to and including narrow bandwidth.

An Al-grating with a Twisted Nematic Liquid Crystal (TN-LC) integrating cell was studied for dynamic color filtering [245]. The cell comprises of Al-grating metasurfaces, fabricated on Indium Tin Oxide (ITO) glass, as a top layer. The bottom plate consists of ITO glass with spin-coated PMMA. The TN-LC was injected via capillary action into the gap between the plates. The TN-LC allows change in the polarization of incident light, while this change can be varied by varying voltage across the plate, hence allowing electrical color tunabiltiy. The idea was further implemented in a an electrically color switching LSPR by exploiting TN-LC [244] (Figure 10B,C).

Here, one of the polarizing plates of TN-LC is replaced by Al-based rectangular nanohole array film; however, an indium tin oxide (ITO) coated glass substrate was used as another electrode. The LC layers formed between both substrates are mutually orthogonal to achieve the TN-LC configuration when light enters the display. Here the asymmetric nanohole array as an output filter allows two different colors in TE and TM mode, and the change in polarization can be induced by applying voltage across TN-LC.

## 6. Conclusions

**Figure 11:**
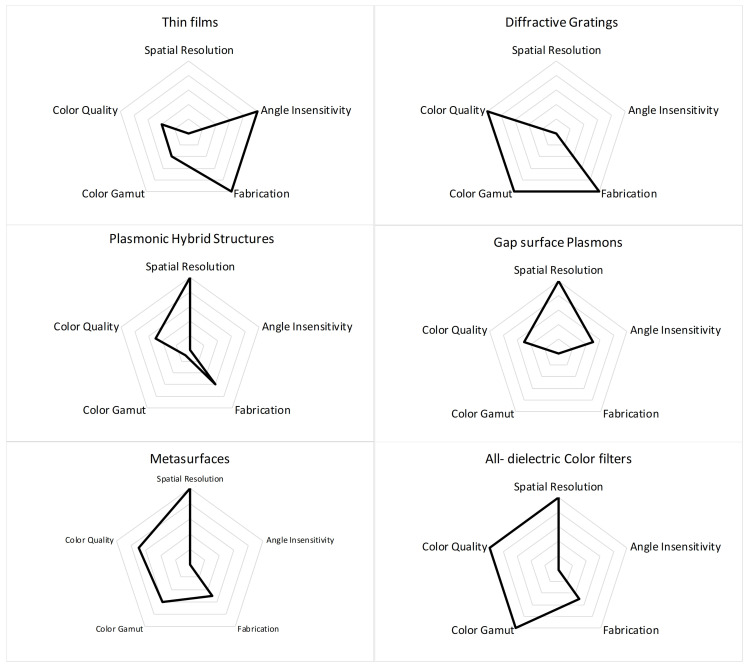
Key Performance Indicator charts for nanostructure color filters.

Ideally, a color filter should have high color quality, a wide gamut, incident angle invariance, high spatial resolution, CMOS compatibility and independence of angle of polarization along with high throughput and cost-effective fabrication techniques. Realistically, a structural color filter with all the above-mentioned qualities does not exist and the replacement of pigment-based color filters is not materialised yet. However, there are examples studied in this review which are exemplary in their own right. So, there is still a need for material exploration and new design technologies to investigate improvements in present designs. This review is further summarized in Figure 11. It gives an overall comparison of key performance indicators of discussed technologies for nanostructure color filters.

From this study, it is inferred that FP-based color filters with lossy materials allow high angle invariance and polarization independence. Furthermore, the FP cavity fabrication requires simple deposition methods and is cost-effective; however, dealing with thin lossy films (proved to have lower losses than its counterparts Au, Ag, and Al) becomes tricky and requires special care. Recently, new methodologies are being adopted to obtain suitable metallic refractive indices such as using the evaporated form of Au/Ag, etc., which has shown efficient results and deserve to be further investigated in the future. In terms of results, generally it has low color quality and limited color gamut and spatial resolution.

Despite of usage of noble metals in the pioneering designs of plasmonic color filter, today Al is preferred over Au, Cu and Ag. This preference is accredited to its compatibility with CIS devices, durability and robustness. Likewise, it is the third most abundant material on earth and also has recycling capabilities.

In general, 1D grating-based structures are prone to viewing angle sensitivity with reasonable color quality. Similarly, it is to be noted that GMR-based color filters produce high quality colors but usually with low spatial resolution. Although it does not require sophisticated fabrication steps, it requires a higher number of gratings and results in a large footprint. Likewise, due to its asymmetric geometry, it is strictly dependent on polarization, but it also makes it a good candidate for polarization tunable color filters.

From LSPRs based structures it can be deduced that nanoholes due to their high angle variance, low spatial resolution and limited color gamut are substituted with advanced structures. The first nanohole-nanodisk hybrid structure employing Fano resonance has paved the way for spatial resolution up to diffraction limit and been an epitome for designs where spatial resolution more than or equal to diffraction limit are desired. Likewise, metasurfaces have shown better performances, but still have lower angle invariance due to LSPRs, which can be further refined by employing novel techniques. Lastly, GSP-based color filters also allowed high angle invariance and spatial resolution with limited color gamut.

Moreover, the advancement in nano-fabrication technologies and simulation methodologies has paved the way for precise and controlled plasmonic-based nanostructures fabrication. Hence, it makes plasmonic-based color filters stand out among their counterparts with high research potential. However, the strong dependence of $\lambda_{res}$ on the device geometry requires a tight critical dimension control especially in fabrication process of metasurfaces, GSP-based color filters and nanohole-nanodisk hybrid structure.

All the studied dielectric-based color filters made of high refractive index (like a-Si,TiO${}_{2}$ and Si${}_{3}$N${}_{4}$ etc.) nano-resonators are studied. It has already sought its due attention because of its compatibility with the CMOS technology. In general, dielectric-based color filters are highly angle sensitive, but it offers high spatial resolution due to high refractive index contrast of nano-resonators and its surroundings. In case of amorphous/crystalline silicon, it offers large gamut coverage, but the color absorption at lower wavelength make it unsuitable for blue colors. Although TiO${}_{2}$ based color filters were demonstrated to avoid absorption for red color (at 650 nm), it invited higher order modes at non-resonant wavelength. Lately, the performance of other dielectric materials Si${}_{3}$N${}_{4}$ based cubic nanostructures showed praiseworthy performance by suppressing higher modes and enhancing the monochromaticity of higher resonant wavelengths by employing Rayleigh anomalies at short wavelength. Hence, pointing towards the research opportunities in this field.

Last section of the review includes LC-based active plasmonic color filters, which allows color tuning in plasmonic structures; this helps to move the $\lambda_{res}$ across the visible regime without any change in its dimensions. This technique has research potential. Therefore liquid crystal with large birefringence, fast switching between states, and its implementation with efficient designs like nanohole-nanodisk hybrid structures and dielectric metamaterials for color filtering, etc., is needs to be explored further.

## Figures and Tables

**Table 1:** Thin Films color filters.

Angle Invariance (${}^{\circ }$)	FWHM (nm)	Mode (T/A/R)	Efficiency (%)	Ref
12	100	T	60	[38]
70	74	T and R	NG	[12]
60	8	T and A	A=97 & T=60	[39]
60	NG	R	NG	[44]
±65	74	R	NG	[43]
60	70	A	NG	[45]
65	100	R	80	[46]
70	NG	T	70	[46]
60	36	T	54	[47]
±60	NA	A	99.58	[48]
50	NG	R	NG	[49]
60	NG	R	90	[50]
30	NA	A	95	[42]

Note: NG = Not given, NA = Not Applicable, A = Absorption, T = Transmission and R = Reflection.

**Table 2:** 1D Diffraction-based color filters.

Angle Invariance (${}^{\circ }$)	FWHM (nm)	Efficiency (%)	Ref
0	30	90	[91]
0	60	73	[93]
0	20	80	[94]
0	12	99	[96]
0	NG	90	[107]
35	NG	95	[98]
0	100	70	[99]
0	17	85	[90]
±80	NG	NG	[100]

Note: NG = Not given.

**Table 3:** LSPR-based color filters.

**Nanohole**				
**Angle Invariance (°)**	**FWHM (nm)**	**Efficiency (%)**	**Spatial Resolution (dpi)**	**Ref**
±20	NG	60–80	NG	[159]
0	NG	14	NG	[163]
70	55	98	NG	[164]
60	145	60	NG	[166]
**Nanohole Hybrid**				
NG	NG	NG	100,000	[167]
±40	NG	NG	141,000	[171]
0	NG	60	77000	[172]
0	NG	NG	127,000	[174]
60	NG	NG	NG	[175]
**Meta surface**				
60	NG	40	100,000	[187]
±8	NG	75	100,000	[191]
60	NG	NG	100,000	[190]
**Gap Surface Plasmon**				
60	110	NG	100,000	[194]
60	NG	NG	100,000	[195]

Note: NG = Not given.

**Table 4:** All Dielectric-based color filters.

Angle Invariance (${}^{\circ }$)	FWHM (nm)	Efficiency (%)	Spatial Resolution (dpi)	Ref.
60	30	64	16,000	[220]
20	NG	NG	85,000	[223]
NG	NG	100	100,000	[227]
26	50–65	Near Zero (A)	NG	[228]
0	NG	90	NG	[222]
0	NG	NG	25,400	[230]

Note: NG = Not given.

## Abbreviations

The following abbreviations are used in this manuscript:

AC	Alternating Current
BSI	Backside Illumination
CCD	Charged coupled Device
CIE	Commission internationale de l’éclairage
CIS	CMOS Image Sensor
CMY	Cyan Magenta Yellow
CMOS	Complementary Metal Oxide Semiconductor
EBL	Electron-Beam Lithography
ED	Electric Dipole
ELT	Extraordinary Low Transmission
EOT	Extraordinary Transmission
FIB	Focused Ion Beam
FWHM	Full Width Half Maximum
GSR	Gap Surface Plasmon
ITO	Indium Titanium Oxide
LC	Liquid Crystal
LCD	Liquid Crystal Display
LSP	Local Surface Plasmon
LSPP	Local Surface Plasmon Polaritons
LSPR	Local Surface Plasmon Resonance
MD	Magnetic Dipole
MIMFP	Metal-Insulator-Metal Fabry-Perot
ND	Nano Disks
NIL	Nanoimprint Lithography
PMMA	Poly Methyl MethAcrylate
RGB	Red Green Blue
SEM	Scanning Electron Microscope
SERS	Surface Enhanced Raman Spectroscopy
SLR	Surface Lattice Resonance
SPR	Surface Plasmon Resonance
sRGB	standarad Red Green Blue
TN	Twisted Nematic
